# Q Fever in Greece and Factors of Exposure: A Multiregional Seroprevalence Study

**DOI:** 10.7759/cureus.69501

**Published:** 2024-09-16

**Authors:** Magdalini K Christodoulou, Konstantinos Tsaras, Charalambos Billinis, Konstantinos I Gourgoulianis, Dimitrios Papagiannis

**Affiliations:** 1 School of Health Sciences, University of Thessaly, Larissa, GRC; 2 Department of Nursing, University of Thessaly, Larissa, GRC; 3 School of Health Sciences, University of Thessaly, Karditsa, GRC; 4 Department of Respiratory Medicine, University of Thessaly, Larissa, GRC

**Keywords:** adults, antibodies, coxiella burnetii, greece, occupational risk exposure, q fever, seroprevalence, vaccination

## Abstract

Introduction: The epidemiology of Q fever, caused by *Coxiella burnetii*, varies significantly worldwide. This study aimed to document the prevalence of *Coxiella burnetii* in Greece by measuring specific IgG antibody levels in serum samples from the general population and high-risk groups, including farmers, veterinarians, and laboratory workers.

Methodology: A multiregional, stratified sampling design was employed, with 1,345 participants from Thessaly and Central Macedonia. Serum samples were tested for *Coxiella burnetii* IgG antibodies, and multivariate analysis was conducted to identify factors associated with seroprevalence.

Results: Overall, 8.1% of participants tested positive for *Coxiella burnetii* antibodies, with the highest seroprevalence in Larissa (22.2%) and Karditsa (16.1%). High-risk occupational groups, particularly those with direct animal contact, showed a higher seroprevalence (13.6%). Multivariate analysis identified significant associations between seroprevalence and factors such as geographic region, occupation, and gender.

Conclusion: The study reveals regional and occupational disparities in Q fever seroprevalence in Greece, particularly in rural areas. These findings underscore the need for targeted public health measures, including heightened surveillance and preventive interventions for high-risk groups.

## Introduction

*Coxiella burnetii*, a gram-negative bacterium, is the causative agent of Q fever, a highly infectious zoonotic disease of global significance, except in New Zealand and Antarctica [1]. This pathogen, which often remains asymptomatic in numerous domestic and wild animal species, can adversely affect reproduction and milk production [2]. During parturition, infected animals excrete large quantities of *Coxiella burnetii*, with the bacterium in high concentrations in the placenta, urine, feces, and milk [3,4]. Human transmission primarily occurs by inhaling infected animals' contaminated dust particles [5,6].

Q fever can present a broad spectrum of severity in humans, from mild flu-like symptoms to life-threatening complications such as pneumonia and endocarditis [7,8]. In 2019, 1,069 cases of Q fever were reported across 23 European Union countries, with 958 (90%) confirmed cases [9]. However, underreporting Q fever cases, especially in countries not previously considered endemic, is a significant issue [10,11]. This poses serious public health challenges and has profound implications for animal and human health [11,12].

*Coxiella burnetii* is mainly transmitted among animals through aerosol dissemination via contaminated dust particles and ingesting infected fetal membranes and fluids during parturition [13]. Humans' primary transmission mode is inhaling contaminated aerosols or dust [14,15]. Additionally, transmission can occur through the gastrointestinal route by consuming contaminated meat, unpasteurized milk, or dairy products [16,17]. There is also a risk of infection for laboratory personnel handling bacterial cultures through percutaneous routes [18]. Human-to-human transmission is rare [19].

Q fever is an occupational disease that affects farmers, abattoir workers, veterinarians, professionals who come into contact with animals, dairy products, animal hides, wool workers, and microbiological laboratory personnel [20,21]. Due to its high infectivity, resistance to many disinfection methods, and long environmental half-life, *Coxiella burnetii* has been classified as a category B bioterrorism agent [22].

From 2004 to 2022, the average annual incidence of Q fever in Greece was 0.06 cases per 100,000 population [23]. The Thessaly region reports the highest incidence, attributed to enhanced laboratory investigation [24]. The disease is often underdiagnosed due to its non-specific clinical presentation and the challenges associated with laboratory confirmation [25]. To address these issues, the CDC has issued national recommendations for Q fever recognition, diagnosis, and treatment, including the management of occupational exposures [26].

Implementing targeted interventions for Q fever is essential due to its potential long-term severe health effects, especially on the cardiovascular system [27]. In Australia, the Q-Vax vaccine is used preventively for high-risk individuals, such as farmers and veterinarians, offering significant protection against the disease [28]. However, in Greece and other European countries, the vaccine is not widely used in humans [29]. Here, prevention mainly relies on the importance of protective measures and the vigilance of monitoring vulnerable groups. This information is crucial for veterinarians and individuals in high-risk occupations, as it helps them feel informed and prepared [30]. Vaccines for animals are available and mainly used in areas with high Q fever rates to prevent abortions and reduce the bacterium's spread. Vaccinating animals helps protect the general population and lowers the risk of transmission to humans [28].

This study is of utmost importance as it aims to conduct a descriptive analysis of data from human serum tests for antibodies against *Coxiella burnetii* in both the general population and high-risk groups in the regions of Thessaly and Central Macedonia. By exploring the relationship between the presence of IgG antibodies and variables such as gender, age, geographic area, animal contact, and occupational exposure, we can provide crucial information for developing effective disease prevention and control strategies. These insights will be instrumental in shaping regional public health policies. According to data from the National Public Health Organization, Thessaly accounted for the highest incidence of Q fever in Greece, representing a staggering 38.7% of reported cases from 2012 to 2022. Although the disease's overall incidence remains low, it may be underreported, particularly in areas that were not previously endemic. This alarming situation presents a significant public health concern with severe animal and human health implications, underscoring the urgency of our research. Therefore, with its precise aim, this study strives to provide valuable insights into the prevalence of *Coxiella burnetii* and robustly support public health efforts to address and prevent Q fever in Greece.

## Materials and methods

Sample calculation and sampling design

The sample size was calculated to be proportional to the general population of the regional units, ensuring a minimum sample size from each regional unit to facilitate reliable comparisons. The population distribution was based on the 2021 census data. The required minimum sample size was determined to be 544 serum samples, calculated using the assumption of an expected prevalence of 50% for *Coxiella burnetii* seropositivity. This is a commonly used approach in epidemiological studies when there is uncertainty or limited prior data regarding the actual prevalence, as it maximizes the required sample size and ensures sufficient statistical power for detecting associations. We used a precision of ±3%, a 95% confidence level, and a power of 80%. This study examined 1,345 serum samples, significantly more than the calculated minimum, to enhance the reliability of the results and reduce potential error in prevalence estimates.

A multiple logistic regression model was developed to assess the independent prognostic factors, examining the effect of gender, age, prefecture, contact with animals, and specific kinds of animals. Variables were included in the multivariate analysis model based on their clinical relevance and statistical significance in the univariate analysis. Only variables with a p-value < 0.1 in the univariate analysis were considered for inclusion in the multivariate model. This approach ensures that the most relevant and potentially confounding variables are evaluated for their independent association with seroprevalence.

The diversity of our sample population was a key strength of our study. Serum samples were collected from a network of health centers in Thessaly and Central Macedonia, including the Amyntaio Health Center in the Florina region. From June 2022 to December 2023, 1,345 blood serum samples were collected. The participants were not limited to the general population but also included individuals belonging to high-risk groups, such as farmers, veterinarians, workers in microbiological laboratories, and health centers. This diverse representation ensured the study's findings were applied to various individuals.

Sample collection

The samples, crucial for our research on *Coxiella burnetii*, were meticulously collected from individuals visiting the health centers in Thessaly and Central Macedonia, including the Amyntaio Health Center in the Florina region, for routine check-ups and other reasons unrelated to *Coxiella burnetii*. Each potential participant was fully informed about the study's objectives and procedures, and their serum samples were anonymized. A unique code was assigned to each sample, and the phlebotomist recorded the required data: gender, age, residence, and date of blood collection. Importantly, all participants provided written informed consent to participate in the research, respecting their autonomy and decision-making. For persons younger than 18 years, consent was obtained from the individual, parent, or legal representative. To ensure the integrity of the serum samples, the blood samples were centrifuged for 10 minutes at 2,500 rpm, and the sera were stored at -20°C. The serum samples, vital for our research, were then transferred to the Public Health and Adult Immunization Laboratory at the University of Thessaly and stored at -80 °C pending analysis. The research protocol was approved by the ethics committee of the University of Thessaly (protocol number: 155/5/20.02.2023) and adhered to the ethical standards described in the Declaration of Helsinki, and all participants provided written informed consent.

Laboratory analysis

The enzyme-linked immunosorbent assay (ELISA) is an important tool for detecting *Coxiella burnetii* antibodies in serum, which helps in diagnosing Q fever. At the Public Health and Adult Immunization Laboratory of the University of Thessaly, we utilized the SERION ELISA classic *Coxiella burnetii* Phase 1 IgG qualitative and quantitative immunoassays from Institute Virion-Serion GmbH, Würzburg, Germany. These assays are specifically designed to detect human antibodies in serum against *Coxiella burnetii*, enabling the laboratory confirmation of Q fever with high sensitivity and specificity. According to the manufacturer's guidelines, values of anti-*Coxiella burnetii* Phase 1 IgG were classified as either <1 IU/mL (negative) or ≥1 IU/mL (positive). We conducted comparisons of the proportions of samples with anti-*Coxiella burnetii* Phase 1 IgG levels in each category based on age group, gender, and region using either the Chi-squared test or Fisher’s exact test.

Statistical analysis

The IgG values were described using the median and IQR, while age was described using the mean and standard deviation. Category variables were described using frequencies and percentages. The Chi-square test, or Fisher’s exact test, was used to identify any association between categorical variables. The statistically significant outcomes were attributed to the use of comparative bar charts. A multiple logistic regression model was developed to assess the independent prognostic factors, examining the effect of gender, age, prefecture, contact with animals, and specific kinds of animals. Variables were included in the multivariate analysis model based on their clinical relevance and statistical significance in the univariate analysis. Only variables with a p-value < 0.1 in the univariate analysis were considered for inclusion in the multivariate model. This approach ensures that the most relevant and potentially confounding variables are evaluated for their independent association with seropositivity. The analysis was conducted using IBM SPSS Statistics software version 26.0 (Armonk, New York, USA).

## Results

The participants had a mean age of 57 years (SD = 19.05) and were drawn from various urban and rural districts within the regions of Central Macedonia and Thessaly, Greece. Table 1 summarizes the demographics, although information on specific professions was not systematically recorded, with 90% of participants not providing this information. Despite this, Table 2 offers detailed data on participants' contact with animal breeding and their classification into high-risk groups. Additionally, Table 3 provides an overview of the types of animals bred by the participants.

**Table 1:** Characteristics of the patients

		Frequency (N)	Percentage (%)
Gender	Male	631	46.9%
	Female	714	53.1%
Profession	Farmer	6	4.9%
	Public employee	1	0.8%
	Slaughterhouse worker	10	8.1%
	Merchant	1	0.8%
	Veterinary pharmaceutical sales representative	1	0.8%
	Health center workers	29	23.6%
	Butcher	5	4.1%
	Veterinarian	2	1.6%
	Breeder	57	46.3%
	Cook	1	0.8%
	Microbiologist	1	0.8%
	Taxi driver	1	0.8%
	Household chores	7	5.7%
	Tailor	1	0.8%
Prefecture	Imathia	238	17.7%
	Thessaloniki	106	7.9%
	Karditsa	87	6.5%
	Kilkis	50	3.7%
	Kozani	45	3.3%
	Larissa	158	11.7%
	Magnesia	66	4.9%
	Pella	216	16.1%
	Pieria	76	5.7%
	Serres	50	3.7%
	Trikala	28	2.1%
	Florina	25	1.9%
	Halkidiki	200	14.9%

**Table 2:** Frequencies and percentages of animal breeding, risk, and positive tests

		Frequency (N)	Percentage (%)
Animal breeding	No	552	41.0%
	Yes	793	59.0%
Risk	High	649	48.3%
	Low	696	51.7%
Test	Negative	1236	91.9%
	Positive	109	8.1%

**Table 3:** Frequencies and percentages of animal breed

	Frequency (N)	Percentage (%) of responses	Percentage (%) of participants
Ruminants	530	36.4%	71.2%
Dogs	386	26.5%	51.9%
Cats	324	22.2%	43.5%
Poultry	149	10.2%	20.0%
Bovines	48	3.3%	6.5%
Pigs	20	1.4%	2.7%
Total	1,457	100.0%	195.8%

**Figure 1:**
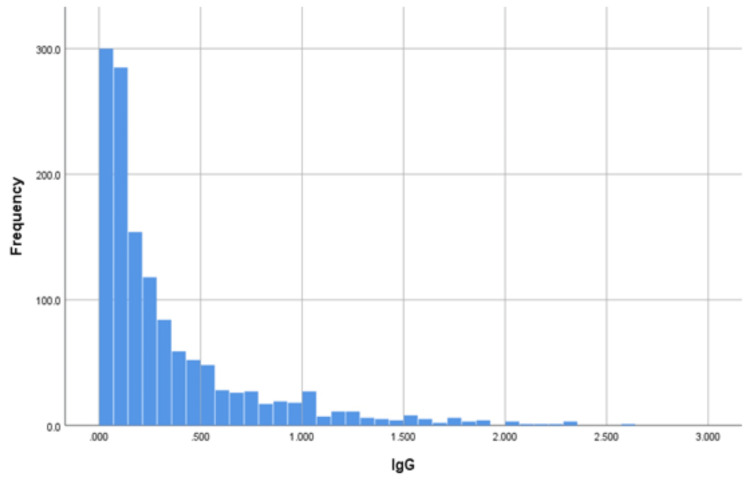
Distribution of the IgG values

Figure 1 illustrates the distribution of IgG values, with a median of 0.18 and an interquartile range (IQR) of 0.10.

In Table 4, the initial analysis revealed statistically significant differences based on geographic area, gender, animal breeding, and belonging to a high-risk group. The only exception was the age category (χ² = 7.967; p = 0.158), where no statistical differences were observed. However, a higher likelihood of a positive test was found in males (χ² = 72.651; p < 0.001), animal breeders (χ² = 33.395; p < 0.001), and high-risk participants (χ² = 114.031; p < 0.001). Certain areas, including Larisa, Karditsa, and Kozani, exhibited a particularly concerning distribution of positive results, with positive tests exceeding 15%. Due to the relatively small number of cases, making clear distinctions in pairwise comparisons between prefectures proves challenging. Accordingly, the grouping based on urbanity revealed higher levels of positive tests in rural areas (χ² = 64.293; p < 0.001).

**Table 4:** Distribution of positive tests by prefecture, urbanity, gender, animal breeding, and risk ELISA: enzyme-linked immunosorbent assay.

		ELISA					
		Negative		Positive			
		Frequency (Ν)	Percentage (%)	Frequency (Ν)	Percentage (%)	χ^2^	p
Prefecture	Imathia	231	97.1%	7	2.9%	72.661	
	Thessaloniki	103	97.2%	3	2.8%		
	Karditsa	73	83.9%	14	16.1%		
	Kilkis	48	96.0%	2	4.0%		
	Kozani	38	84.4%	7	15.6%		
	Larissa	123	77.8%	35	22.2%		
	Magnesia	61	92.4%	5	7.6%		
	Pella	203	94.0%	13	6.0%		
	Pieria	68	89.5%	8	10.5%		
	Serres	48	96.0%	2	4.0%		
	Trikala	27	96.4%	1	3.6%		
	Florina	23	92.0%	2	8.0%		
	Halkidiki	190	95.0%	10	5.0%		
Age group	<18	57	98.3%	1	1.7%	7.967	0.158
	18-30	80	96.4%	3	3.6%		
	31-40	111	94.1%	7	5.9%		
	41-50	174	91.6%	16	8.4%		
	51-60	246	91.8%	22	8.2%		
	>60	568	90.4%	60	9.6%		
Urbanity	Urban	377	99.5%	2	0.5%	64.293	
	Semiurban	451	93.2%	33	6.8%		
	Rural	408	84.6%	74	15.4%		
Gender	Male	551	87.3%	80	12.7%	33.395	
	Female	685	95.9%	29	4.1%		
Animal breeding	No	551	99.8%	1	0.2%	78.915	
	Yes	685	86.4%	108	13.6%		
Risk group	High	543	83.7%	106	16.3%	114.031	
	Low	693	99.6%	3	0.4%		

Table 5 presents the results of the Chi-square analysis, indicating that breeding bovines (39.6%, n = 19), sheep (16.0%, n = 85), or poultry (14.8%, n = 22) significantly raises the likelihood of a positive test outcome. However, the finding concerning dogs (11.4%, n = 44) is considered confounding, as breeders usually have dogs, although not pets.

**Table 5:** Chi-square analysis: distribution of positive tests by breeding animals ELISA: enzyme-linked immunosorbent assay.

		ELISA					
		Negative		Positive			
		Frequency (Ν)	Percentage (%)	Frequency (Ν)	Percentage (%)	χ^2^	p
Small ruminants	No	791	97.1%	24	2.9%	73.924	
	Yes	445	84.0%	85	16.0%		
Dogs	No	894	93.2%	65	6.8%	7.892	0.005
	Yes	342	88.6%	44	11.4%		
Cats	No	940	92.1%	81	7.9%	0.166	0.684
	Yes	296	91.4%	28	8.6%		
Poultry	No	1,109	92.7%	87	7.3%	9.983	0.002
	Yes	127	85.2%	22	14.8%		
Bovines	No	1,207	93.1%	90	6.9%	66.233	
	Yes	29	60.4%	19	39.6%		
Swine	No	1,220	92.1%	105	7.9%	3.858	0.072
	Yes	16	80.0%	4	20.0%		

In Table 6, the binary logistic regression model results show that gender, high-risk group status, animal breeding, bovine breeding, and urbanity are independent prognostic factors for testing positive. Specifically, the likelihood of males testing positive was 3.04 times higher than that of females (OR = 3.04; 95% CI: 1.91-4.84; p < 0.001). High-risk participants had 9.61 times greater odds of testing positive than low-risk participants (OR = 9.61; 95% CI: 2.74-33.68; p < 0.001). Bovine breeders had 3.45 times greater odds of testing positive than non-bovine breeders (OR = 3.45; 95% CI: 1.79-6.66; p < 0.001), and animal breeders had 9.18 times greater odds of testing positive than non-animal breeders (OR = 9.18; 95% CI: 1.09-77.43; p = 0.042). Additionally, the odds of testing positive in rural locations were 5.73 times higher than in urban locations (OR = 5.73; 95% CI: 1.33-24.63; p = 0.019). Semi-urban locations did not exhibit significant differences in odds compared to urban or rural locations. The model explained 32.4% of the variance in testing positive, as indicated by the Nagelkerke R square value.

**Table 6:** Binary logistic regression analysis of independent prognostic factors for testing positive

							95% CI for OR	
Variables in the equation				df	Sig.	OR	Lower	Upper
Age	-0.004	0.006	0.468	1	0.494	0.996	0.983	1.008
Male vs female	1.111	0.238	21.835	1	0.000	3.038	1.906	4.841
High vs low risk	2.262	0.640	12.496	1	0.000	9.606	2.740	33.677
Cow breeding	1.239	0.335	13.653	1	0.000	3.452	1.789	6.662
Animal breeding	2.217	1.088	4.153	1	0.042	9.180	1.088	77.430
Semi-urban vs rural	1.414	0.755	3.508	1	0.061	4.113	0.936	18.065
Urban vs rural	1.745	0.744	5.495	1	0.019	5.725	1.331	24.628
Constant	-6.471	1.352	22.910	1	0.000	0.002		

In Table 7, the comparison is made between the arithmetic and geometric means of the ELISA values. The arithmetic mean is derived by summing the values and dividing by the number of observations, providing an average. In contrast, the geometric mean is obtained by multiplying the values and taking the nth root, where n represents the number of observations. The geometric mean offers a more balanced representation of the entire dataset and is less influenced by extreme values, particularly accentuating the significance of more minor observations. This characteristic makes it particularly advantageous in scenarios involving negative ELISA values, where it can illuminate differences that may not be readily apparent when relying on the arithmetic mean, especially when the two mean values are closely aligned.

**Table 7:** Comparison of arithmetic and geometric means for ELISA results ELISA: enzyme-linked immunosorbent assay.

ELISA	Mean	Geometric mean	N
Negative	0.2458	0.1624	1,235
Positive	1.3963	1.3527	109
Total	0.3391	0.1929	1,344

**Figure 2:**
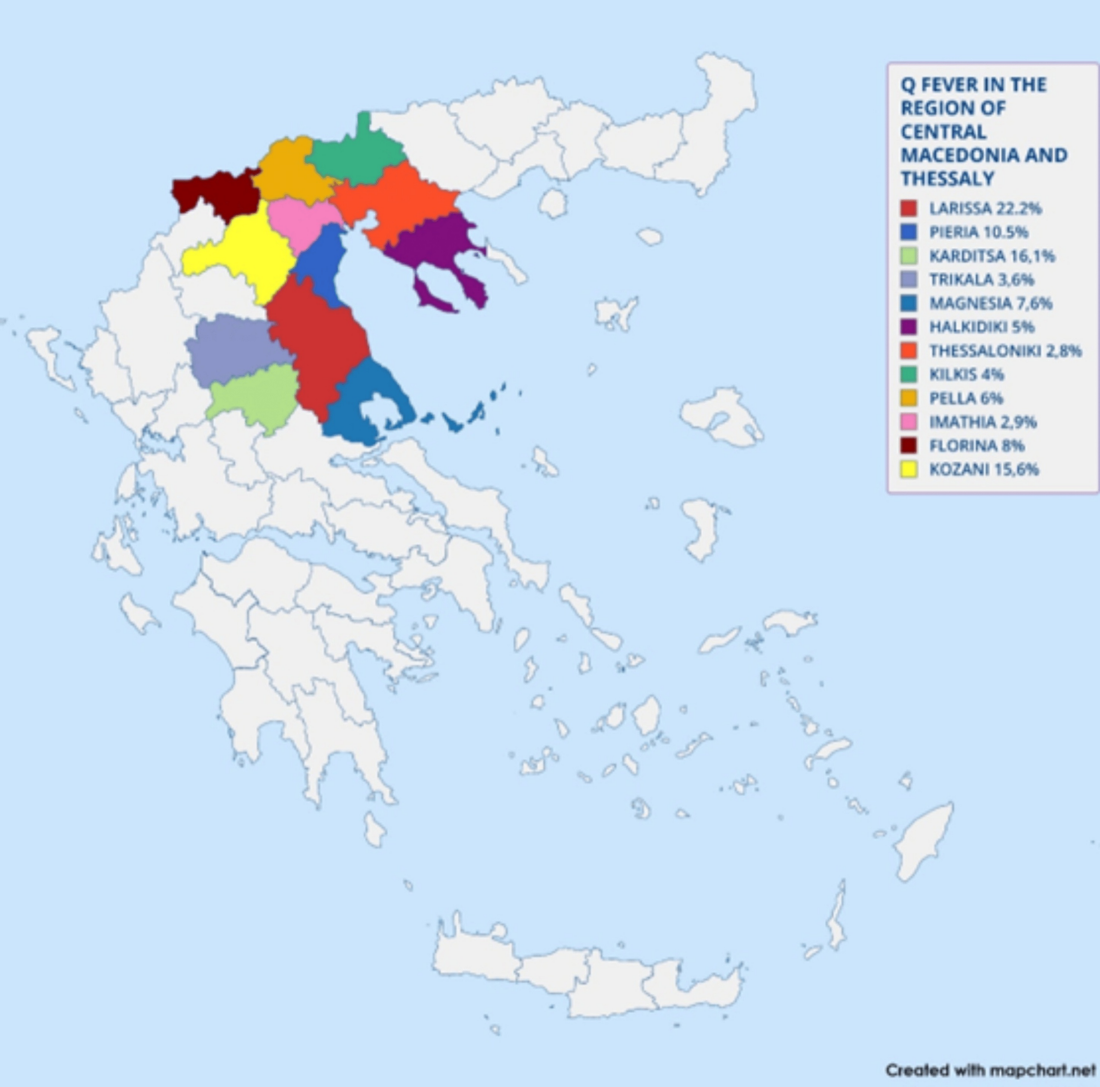
Seroprevalence of Q fever in the region of Central Macedonia and Thessaly, Greece

As shown in Figure 2, the seroprevalence of Q fever varies significantly across different regions, with the highest rates observed in Larissa (22.2%) and Karditsa (16.1%).

Gender

The study revealed that males had a significantly higher likelihood of testing positive for *Coxiella burnetii* than females. Males were found to have 3.04 times higher odds (OR = 3.04; 95% CI: 1.91-4.84; p < 0.001). The positive rate was 12.7% (n = 80/631) for males and 4.1% (n = 29/714) for females.

Age

Differences in prevalence based on age were observed. The highest positive rates were seen in the age groups 51-60 years (8.2%, n = 22/268) and over 60 years (9.6%, n = 60/627). However, the Chi-square test did not find significant differences across age groups (χ² = 7.967, p = 0.158).

Prefecture

Significant regional differences were found, with the highest prevalence in Larissa (22.2%, n = 35/158), followed by Karditsa (16.1%, n = 14/87) and Kozani (15.6%, n = 7/45). Other regions had lower prevalence rates, such as Thessaloniki (2.8%, n = 3/106) and Imathia (2.9%, n = 7/238) (χ² = 72.661, p < 0.001).

Urbanity

Prevalence was significantly higher in rural areas (15.4%, n = 74/481) compared to urban (0.5%, n = 2/400) and semi-urban regions (6.8%, n = 33/484) (χ² = 64.293, p < 0.001). Rural residents had 5.73 times higher odds of testing positive than urban residents (OR = 5.73; 95% CI: 1.33-24.63; p = 0.019).

## Discussion

The results of our study highlight the substantial regional differences in *Coxiella burnetii* seroprevalence across Greece, with the highest prevalence observed in Larissa (22.2%) and Karditsa (16.1%). Additionally, individuals with direct animal contact had significantly higher seroprevalence (13.6%) compared to those without (0.2%), and high-risk occupational groups showed a positive rate of 16.3%. These findings underscore the importance of targeted interventions in these high-risk groups and regions. Despite the implementation of global control measures, Q fever remains a significant public health challenge, as evidenced by the varying prevalence rates observed in different regions [31]. Data from the European Centre for Disease Prevention and Control (ECDC) indicates that Greece has low incidence rates of Q fever in the general population and specific high-risk groups [24,32-34]. Our study is the first to examine the prevalence of *Coxiella burnetii* infection in the general adult population in Thessaly and Central Macedonia, contributing to the global understanding of this zoonotic disease. The results of our study revealed a low occurrence of anti-*C. burnetii* Phase I IgG (>1 IU/mL) in the general population. The prevalence rates of *Coxiella burnetii* varied widely, ranging from 2.8% (n = 3/106) in Thessaloniki to 22.2% (n = 35/158) in Larissa. This variation aligns with other studies, such as those conducted in Portugal, where the seroprevalence was 5.8% during 2010-2013, and the incidence ranged from 0.1 to 2.2 per 100,000 citizens [35]. These findings, alongside high notification rates among livestock handlers and individuals in rural areas, underscore the significant underreporting of Q fever cases among the general adult population and highlight the crucial role of health professionals in addressing this issue. This issue is not confined to Portugal but is also prevalent in Australia [36], the United States [37], and France [38], emphasizing the need for increased awareness and a broader use of serological testing to enhance the diagnosis and reporting of Q fever cases. Our data indicated significant regional variations in the prevalence of *Coxiella burnetii* antibodies. Larissa exhibited the highest prevalence at 22.2% (n = 35/158), followed by Karditsa at 16.1% (n = 14/87) and Kozani at 15.6% (n = 7/45). In contrast, Thessaloniki, Imathia, Trikala, and Serres displayed lower prevalence rates of 2.8% (n = 3/106), 2.9% (n = 7/238), 3.6% (n = 6/167), and 4% (n = 6/150), respectively. Differences may influence these variations in the population's immune status and sample collection methods. For instance, samples from Larissa included representation from many rural villages, while those from Thessaloniki, Imathia, and Kilkis were primarily collected from urban areas and private microbiological laboratories. Comparing our findings with international data, studies in Italy reported a high prevalence of *C. burnetii* antibodies (44.0%) among agricultural workers, with rates ranging from 2.8% to 75.9%, indicating significant underreporting of *C. burnetii* infections in the country [39]. Similarly, other studies found varying prevalence rates of *Coxiella burnetii* antibodies in different regions, such as 5% in the general population of Tuscany and 48.6% in Eastern Cantabria, Spain, reporting a prevalence of 48.6%, with higher rates in older individuals and rural areas [39]. Additionally, a study in the Basque country observed a higher prevalence in sparsely populated areas and among individuals with agricultural/livestock exposure [40]. Our study further confirmed that males are more likely to test positive for *Coxiella burnetii* antibodies, with a positive rate of 12.7% (n = 80/631) compared to 4.1% (n = 29/714) in females. Studies have consistently found a higher prevalence of *Coxiella burnetii* antibodies in males than in females. For instance, Bartolomé et al. reported a seropositivity rate of 29% in men compared to 18% in women [41], while Saz et al. found rates of 32.7% in men and 8.8% in women [42]. Vilibić-Čavlek et al. also observed a higher seroprevalence in men (31.6%) compared to women (22.2%) [43]. The observed gender differences in *Coxiella burnetii* seroprevalence may be partly attributed to increased occupational exposure among males. Studies have shown that occupational exposure to livestock is a significant risk factor for Q fever. In our study, individuals with direct animal contact had a seroprevalence rate of 13.6% (n = 108/793), significantly higher than those without such contact (0.18%, n = 1/552). High-risk occupational groups, such as farmers and veterinarians, had a positive rate of 16.3% (n = 106/649), further underscoring the role of occupational exposure in the epidemiology of Q fever. It is essential to ascertain whether the observed difference pertains to infection rates or the illness's severity and complications. According to Lamas et al. (2009), the prevalence of *Coxiella burnetii *antibodies was similar in both females and males, suggesting no disparity in susceptibility to infection [44]. A recent study investigated the frequency and causes of previous *Coxiella burnetii* (Q fever) infections in Northern Ireland. The study analyzed serum samples from 2,394 individuals aged 12-64, collected during surveys in 1986 and 1987. The overall prevalence of *Coxiella burnetii *antibodies was 12.8%, with a slightly higher prevalence in males compared to females (14.3% vs. 11.2%). Seropositivity was low in children but increased in the 25-34 age group and remained consistent with age. Farmers had a significantly higher seropositivity rate (48.8%) than the general population. Furthermore, seropositive women experienced a higher frequency of miscarriage or stillbirth (19.5% vs. 9.8%) [45]. The difference in *Coxiella burnetii* seroprevalence between genders may be partly attributed to the increased occupational exposure to livestock among males compared to females [46]. Studies have shown that occupational exposure is a significant risk factor for Q fever [47]. In our study, individuals with direct animal contact had significantly higher seroprevalence rates (13.6%) than those without contact (0.18%). High-risk occupational groups, such as farmers and veterinarians, showed a positive rate of 16.33%. These findings are consistent with international data, highlighting the crucial role of occupational exposure in the epidemiology of Q fever [48]. Specifically, the role of bovine exposure is critical. Our study found that individuals involved in bovine breeding had a significantly higher prevalence of *Coxiella burnetii* antibodies than those not involved with cattle. This is consistent with findings from other regions. For instance, a study in the Netherlands from 2010 to 2011 found that 72.1% of residents and workers on large dairy cattle farms had antibodies against *Coxiella burnetii*, indicating a high prevalence of Q fever. The prevalence was 87.2% among farmers, 54.5% among spouses, and 44.2% among children. Identified risk factors included farm location in the southern region, larger herd size, employment on the farm, the presence of birds in the stable, contact with pigs, and indirect contact with rats or mice. Protective factors included using automatic milking systems and consistent use of gloves during calving [49]. Our findings also revealed that the odds of being seropositive were significantly higher in rural areas compared to urban areas, with rural residents having 5.73 times greater odds of being seropositive. This is consistent with studies from other regions, such as Eastern Cantabria and the Basque country in Spain, where higher prevalence rates were observed in rural and sparsely populated areas [50]. The higher seroprevalence rates in rural areas can be attributed to several factors, including greater exposure to livestock, environmental contamination, and limited healthcare services. Livestock farming is more prevalent in rural areas, increasing the risk of exposure to *Coxiella burnetii* through direct contact with infected animals or contaminated environments [51]. The lower access to healthcare services in rural areas may result in delayed diagnosis and underreporting of Q fever cases, contributing to the observed differences in seroprevalence [52]. High-risk groups are of particular interest in the epidemiology of Q fever [53]. In South Korea, a significant increase in Q fever cases was observed in 2015. A study of 661 workers in the veterinary service laboratory revealed a seroprevalence of 7.9% and a seroreactivity of 16.0% for *Coxiella burnetii* infection. Occupational exposure to livestock and the nature of veterinary work likely contribute to the higher prevalence rates observed in this group [54,55]. In 1952, Iran reported its first cases of acute Q fever. From 1970 to 1976, 133 cases were documented in various parts of the country. No human cases were reported after 1976 until 2013, when the first chronic Q fever (endocarditis) was documented [56].

Strengths

This study is the first multiregional seroepidemiological investigation of *Coxiella burnetii* exposure among the general population in the regions of Thessaly and Central Macedonia. A key strength of this study lies in its geographically stratified sampling design, which ensured representation across different regions, age groups, genders, and occupational categories. By collecting data from both rural and urban areas, the study provides a comprehensive overview of the seroprevalence of *Coxiella burnetii* in these regions. Furthermore, the inclusion of high-risk occupational groups, such as farmers and veterinarians, enhances the relevance of the findings for public health planning.

Limitations

However, the study also has several limitations. While geographically broad, the scope of the study was limited to Thessaly and Central Macedonia, which may not be fully representative of the broader Greek population. Additionally, the cross-sectional design prevents the establishment of temporal relationships between exposure to *Coxiella burnetii* and the development of clinical manifestations. The reliance on self-reported data for risk factors, such as animal contact, introduces the possibility of recall bias. Finally, the potential for selection bias exists due to varying response rates across regions and age groups. Despite these limitations, our study, the first multiregional seroepidemiological study of *Coxiella burnetii* exposure among the general population in the regions of Thessaly and Central Macedonia, has yielded significant findings. While not comprehensive, these findings offer valuable insights for policymakers and health professionals, empowering them to shape future regional public health strategies and campaigns for the prevention and vaccination against *Coxiella burnetii.*

## Conclusions

This study offers significant insights into the epidemiology of Q fever in Greece, highlighting regional, gender, age, and occupational differences in *Coxiella burnetii* seroprevalence. The highest prevalence rates were observed in Larissa (22.2%) and Karditsa (16.1%), which may be linked to specific occupational exposures and environmental factors, although these associations warrant further investigation. Previous studies suggest that environmental contamination, especially in rural areas, and close contact with animals are major contributors to the spread of *Coxiella burnetii*. Our findings indicate that despite existing control measures, herd immunity remains insufficient, particularly among high-risk occupational groups such as farmers and veterinarians. Targeted public health interventions are crucial for reducing the transmission of *Coxiella burnetii*. Proper hygiene practices, management of contaminated materials (e.g., manure treatment and safe disposal of animal carcasses), and vaccination are essential strategies. The Q-Vax vaccine, which has demonstrated 98% efficacy in Australia, is a promising tool for enhancing prevention efforts in high-risk populations. Furthermore, increasing awareness, improving serological testing, and expanding vaccination coverage are essential components of a comprehensive approach to Q fever control in Greece. These findings highlight the urgent need for policymakers and healthcare professionals to implement enhanced public health strategies to reduce the burden of Q fever.
